# Medical education and informal teaching by nurses and midwives 

**DOI:** 10.5116/ijme.53f5.ee77

**Published:** 2014-08-31

**Authors:** Jean Gilmour, Annette Huntington, Fiona Bogossian, Bernadette Leadbitter, Catherine Turner

**Affiliations:** 1 School of Nursing, Massey University, New Zealand; 2 School of Nursing & Midwifery, The University of Queensland, Queensland, Australia

**Keywords:** Medical education, nurses, midwives, survey, informal teaching

## Abstract

**Objectives:**

The aim of this study was to examine the contribution of nurses and midwives to the education of medical colleagues in the clinical context.

**Methods:**

The research design was a cross-sectional survey using an online questionnaire. A subsample of 2906 respondents, from a total of 4763 nurses and midwives participating in a web-based study, had taught doctors in the 12 months prior to the survey. The questionnaire generated mainly categorical data analysed with descriptive statistics.

**Results:**

In the group of respondents who taught doctors (n =2906), most provided informal teaching (92.9%, n=2677). Nearly a quarter (23.9%, n=695) self-rated the amount of time spent teaching as at least moderate in duration. The most common named teaching topics were documentation (74.8%, n=2005) and implementing unit procedures (74.3, n=1987), followed by medication charting (61.9%, n=1657) and choosing correct medications (55.8%, n=1493). Respondents felt their contributions were unrecognised by the doctors and students they taught (43.9%, n=1256).

**Conclusions:**

Educational contributions while unrecognised could be considered positively by the respondents. However, discussion of teaching responsibilities is necessary to support the development of teaching protocols and supervision responsibilities as respondents reported teaching clinical medical tasks related to medications, consent and other skills within the medical domain. Study limitations include the nature of self-reported responses which cannot be validated and data drawn from a survey concluded in 2009.

## Introduction

Nurses and midwives work closely with medical staff in all aspects of health care delivery and have a substantial role in teaching junior medical staff in the clinical environment as a part of their contribution to the work of the health care team. The sharing of knowledge in the multidisciplinary team benefits patients and promotes productive work relationships. However, there appears to be little formal recognition that teaching junior medical staff in the clinical environment is a team enterprise.A review of the international literature indicates that nursing contributions to medical education in the clinical environment is under researched and poorly understood. Two studies explicitly explore informal nursing education roles with medical staff.^1^^,^^2^ In an Australian qualitative study^1^ nurses and interns were interviewed about nursing teaching activities. Nurses in the study discussed interns’ knowledge gaps about hospital protocols and clinical procedures. The need to teach doctors in order to maintain patient safety was complicated by roles and expectations. Vallis et al.^2^ studied Scottish senior nurses and the informal training of pre-registration house officers. Nurses in this study reported a desire to formalize their teaching roles in order to recognise their experience and expertise. Other research studies on nursing roles identified nurse practitioners who informally teach junior doctors in medical wards^3^ and nurses in general practice who teach medical staff.^4^The role of midwives in medical education is more fully documented in the literature with a complete issue of the Journal of Midwifery & Women’s Health in 2009 focused on the contribution of midwives to medical education (Volume 54, Issue 4). An American medical school survey completed in 1995 identified that half of the 129 schools who responded used midwives as educators. In this study 176 of the nurse-midwives’ reported they were normally involved in medical student education.^5^ A more recent American study building on the earlier study, with responses from 74 midwifery practices, found the majority taught residents and medical students as well as midwifery students.^6^ Some midwives’ reported that they did not take midwifery students because of medical teaching obligations. Australian research on extended practice roles also found that midwives’ described teaching new doctors about midwifery practise and birthing.^7^ Quinlivan, Black, Petersen and Kornman^8^ stated that midwives frequently supervise and teach medical students in a relationship that is informally recognised but without specific educational preparation.There is little research evidence that documents the contribution by nurses to medical staff education. By comparison, the acknowledgment of midwives’ contribution is more explicit in the literature with American surveys quantifying the extent of teaching contribution. A cohort study of nurses’ work-life and health in Australia, the UK and NZ, provided the opportunity to gather further data on the contribution of nurses and midwives to medical education. Consequently, this study aimed to examine the characteristics of nurses and midwives involved in informal medical teaching, the extent and form of the teaching, and the topics taught in order to identify any potential legal or regulatory issues.

## Methods

### Design

The study used a short questionnaire delivered as part of the second survey of the longitudinal web-based study, The Nurses and Midwives e-cohort Study (NMeS). The study focuses on work-life balance and staying healthy. The study design is described in detail in previous publications.^9^^,^^10^ Nurses and midwives from Australia, NZ and the UK were invited to be part of the longitudinal study from April 2006 to March 2008. The study was advertised through the New Zealand and Australian Nursing Councils and other media. There were variable recruitment methods across jurisdictions.^9^ The questions generating the data discussed in this paper were part of the second survey carried out between August 2008 and September 2009.The study (Australia, NZ and UK) was approved by the Behavioural and Social Sciences Ethical Review Committee of The University of Queensland (No.2005000696). The NZ component was approved by the Massey University Human Ethics Committee (Wellington: No.05/71).

### Sample and sample size

A total of 4763 respondents participating in the NMeS, and employed as registered nurses and midwives at the time of the second survey, answered the questions on teaching activities with medical staff. Respondents who identified that they were not working at the time of the survey in nursing and midwifery were excluded, along with enrolled nurses, nurse aides and student nurses. A subsample of 2906 nurses and midwives who had taught medical staff in the last 12 months were extracted from the total sample.

### Data collection

The questionnaire included questions focused on nurses and midwives providing clinical teaching to doctors and medical students along with questions about the demographic characteristics of the sample (age, qualification, education, country of registration and employment classification). The questions were developed drawing on the expertise of the nursing and midwifery investigators with subsequent review by the wider team of NMeS investigators from a range of disciplines. These questions were then piloted with nurses and midwives as part of the processes adopted for the second survey of the longitudinal study to establish face validity, and check the overall question flow and clarity.Most questions (n = 6) generated dichotomous yes or no responses. All respondents were asked if they thought formal teaching of doctors was part of the nursing and midwifery role and if they had taught doctors or medical students in the last 12 months. The group that had taught in the last 12 months were then asked how that teaching was recognised, the topics they taught, and their perception of the amount of time they spent teaching. A scale from ‘a little’ or ‘no time’ to ‘moderate’ or ‘a lot’ was used. Participants were not personally identified in the analysis datasets.

### Data analysis

The descriptions of the sample and subsample who taught doctors and medical students in the 12 months preceding the survey are reported using summary statistics; mean for continuous variables, and counts/percentages for categorical variables. The Pearson’s χ^2^ test was used for categorical tests of proportions and the means between groups are compared using the Student’s t-test. SPSS 17.0 (IBM SPSS Inc., Chicago, IL, USA) for Windows was used for the statistical analysis. Reponses to the questions were voluntary and the total responses vary by question.

## Results

The majority of respondents reported that the formal teaching of doctors was part of a nursing/midwifery role (66.6%, n=3155), and 79.2% (n=3761) worked with doctors or medical students in clinical settings (Table 1).

**Table 1:** Formal and informal teaching (N = 4746)

Teaching	Answer	n	%
Formal teaching of doctors as part of nursing/midwifery role	Yes	3155	66.6
	No	1583	33.4
	Total	4738	
Work with doctors/medical students in clinical setting	Yes	3761	79.2
	No	985	20.8
	Total	4746	
Teaching of doctors/medical students in last 12 months	Yes	2906	77.1
	No	865	22.9
	Total	3771	
Teaching done informally on the job	Yes	2677	92.9
	No	206	7.1
	Total	2883	
Was teaching over the last 12 months in formal sessions?	Yes	415	14.4
	No	2467	85.6
	Total	2882	

There was a significant association between the perception that formal teaching was part of the nursing and midwifery role and qualification level (χ^2^_(1)_ =86.865, p=<0.001). Just under three-quarters of respondents with postgraduate qualifications (72.5%, n=1851) agreed that formal teaching was part of the role compared to 59.7% (n=1303) of respondents with qualifications to degree level. The mean age of respondents agreeing that formal teaching was part of the role (45.18 years, n=3155) was significantly higher than those who did not agree (43.25 years, n=1583) (t_(3010)_= - 6.583, p=<0.001); the Levene's test result was statistically significant (P=.000).A smaller group of respondents had taught medical staff in the last 12 months (n=2906) making up 61% of the total sample and 77.1% of the group who worked with medical staff in clinical settings. The demographic characteristics of the overall sample and the subsample are detailed in Table 2. The sample and subsample are described by employment qualification and country of registration in Table 3.

**Table 2:** Demographic characteristics of sample of employed nurses and midwifes (N=4763) and subsample who had taught medical staff in last 12 months (N=2906)

Variable		Total Sample N=4763		Subsample (taught medical staff in last 12 months) N=2906	
		n	%	n	%
Age	Range	19-73 years		20-73 years	
	Number	4763		2906	
	Mean (SD)	44.5 (9.4)		43.8 (9.2)	
Gender	Female	4377	91.9	2667	91.8
	Male	386	8.1	239	8.2
Highest professional qualification	Certificate	697	14.7	405	13.9
	Diploma	307	6.4	169	5.8
	Bachelor Degree	1190	25	705	24.3
	Honours Degree	259	5.4	172	5.9
	Post Grad Cert	723	15.2	481	16.6
	Post Grad Dip	824	17.3	524	18
	Masters	699	14.7	425	14.6
	PhD	61	1.2	23	0.8
Position	Registered Nurse	2199	46.3	1299	44.8
	Registered Midwife	309	6.5	221	7.6
	Registered Nurse and Midwife	204	4.3	146	5.0
	Clinical Nurse Specialist	595	12.5	450	15.5
	Clinical Midwifery Specialist	83	1.7	70	2.4
	Nurse Practitioner	108	2.3	93	3.2
	Clinical Educator	164	3.5	104	3.6
	Unit Manager	287	6.0	194	6.7
	Tertiary Lecturer	138	2.9	30	1.0
	Administrator/ Manager	258	5.4	80	2.8
	Other	407	8.5	214	7.4

**Table 3:** Employment qualification and country of registration council by total sample (N = 4727) and subsample (N = 2887)

Country of registration council	Total Sample						Subsample (taught medical staff in last 12 months)					
	Nurse N=3909		Midwife N=403		Nurse/ Midwife N=415		Nurse N=2324		Midwife N=304		Nurse/ Midwife N=259	
	n	%	n	%	n	%	n	%	n	%	n	%
Australia	2244	57	247	61	384	93	1314	57	191	63	240	93
New Zealand	784	20	8	2	11	2	447	19	7	3	6	2
UK	834	21	143	35	18	4	531	23	101	33	12	5
Other	47	1	5	1	2	1	32	1	5	2	1	0

Analysis of the subsample of nurses and midwives who taught doctors and medical students in the last 12 months found the majority provided informal education (92.9%, n =2677). A smaller proportion (14.4%, n=415) provided formal teaching sessions. A significantly higher proportion of midwives were engaged in teaching medical staff in the last 12 months (84.7%, n=305), compared to nurses (75.9%, n=2342), and those employed as both nurse and a midwife (79.4%, n=259) (χ^2^_(2)_ =15.293, p <0.001).

In the subsample group, 20.8% (n=604) rated the amount of time spent in teaching activities as ‘moderate’ and 3.1% (n=91) as ‘a lot’, for the majority the time was judged as ‘a little’ (74.7%, n=2172). There was no significant relationship between employment as a nurse, midwife or both, and the time spent teaching medical staff as categorised as either a little to no time, or moderate to a lot of time. There was also no significant association between the perception that formal teaching was part of the nursing and midwifery role and time spent teaching.However, higher educational levels were significantly associated with time spent teaching (χ^2^_(1)_=12.455, p=<0.001) with 26.4% (n=430) of nurses and midwives with postgraduate qualifications spending at least a moderate amount of time teaching medical staff compared to 20.8% (n=265) of those with qualifications to degree level. More experience as a midwife was also significant. Midwives reporting moderate or more time teaching had a mean of 14.5 years of experience (n=186), while those reporting little time spent teaching had a mean of 11.9 years of experience (598) (t_(782)_=-3.120, p=<0.002). Experience was not a differentiating factor in nurses’ time spent teaching with a mean of 19.2 years of experience (n=683) for those reporting a moderate amount or more and a mean of 19.7 years (n=2158) for those reporting little time spent teaching.The proportions of particular topics informally taught by nurses and midwives are detailed in Table 4. Teaching medical staff about documentation (74.8%, n=2005) and implementation of ward/unit procedures (74.3%, n=1987) were the most common specific content teaching areas, followed by medication charting (61.9%, n=1657), and choosing correct medications (55.8%, n=1493). There was little formal acknowledgement of teaching contribution, 43.9% (n=1256) of the respondents felt their contribution was unrecognised by the doctors and students they taught (Table 4).

**Table 4:** Informal teaching topics (N = 2679) and recognition of teaching role (N = 2864)

Teaching topics				Recognition of teaching role			
		n	%			n	%
Charting medications	Yes	1657	61.9	Time allowance/in lieu	Yes	119	4.2
	No	1018	38.1		No	2745	95.8
	Total	2675			Total	2864	
Choosing correct medications	Yes	1493	55.8	Financial payment to the nursing/midwifery budget	Yes	57	2
	No	1181	44.2		No	2802	98
	Total	2674			Total	2859	
Completing documentation	Yes	2005	74.8	Recognition at ward level by senior staff	Yes	529	18.6
	No	674	25.2		No	2316	81.4
	Total	2679			Total	2845	
Inserting IV lines/cannulas	Yes	685	25.7	Individual recognitionfrom the doctor(s)/student(s) taught	Yes	1605	56.1
	No	1982	74.3		No	1256	43.9
	Total	2667			Total	2861	
Inserting chest drains	Yes	99	3.7	Another method	Yes	120	4.3
	No	2571	96.3		No	2662	95.7
	Total	2670			Total	2782	
Inserting urinary catheters	Yes	460	17.3	Not recognised	Yes	1353	48.5
	No	2202	82.7		No	1438	51.5
	Total	2662			Total	2791	
Implementation of ward/unit procedures	Yes	1987	74.3				
	No	688	25.7				
	Total	2675					
Getting patient consent	Yes	1106	41.4				
	No	1568	58.6				
	Total	2674					
Taking blood	Yes	670	25.1				
	No	1996	74.9				
	Total	2666					
Other clinical, ward or patient skills	Yes	2141	80.1				
	No	533	19.9				
	Total	2674					

## Discussion

The majority of nurses and midwives in this study reported that they contributed to medical education in areas where they had expertise. One-fifth reported that they spent a moderate to a lot of time on medical staff education. Overwhelmingly the teaching contribution was informal and recognition was limited with a very small group receiving a time allowance or financial remuneration to the nursing or midwifery budget for the extra responsibility. These findings are consistent with previous research.^8^^,^^11^ The majority of respondents stated that they believed collegial teaching was part of their role, and agreement was significantly associated with advanced education and older age. These findings suggest that postgraduate nursing preparation supports the readiness of nurses and midwives, and possibly their feelings of obligation, to teach other health professionals.The most common specified teaching topics in this study were about unit procedures and documentation, which are part of professional orientation and enculturation. A considerable percentage of respondents also reported teaching clinical medical tasks related to medications, consent and other skills. It is also implied in the literature that junior doctors or medical students might seek out advice of a senior nurse in relation to certain procedures, or medication advice. Pearson et al.^12^ examined factors which influence intern prescribing. The results from the qualitative study confirmed registrars as being the most approachable for advice; however, they did acknowledge nurses’ experience and guidance in prescribing medications. The study also identified nurses and pharmacists as “under- utilized resources for formal and opportunistic prescribing teaching”. Jutel and Menkes^13^ used a web-based survey to identify any influences a group of senior nurses might knowingly or unknowingly have on the decision making of the prescriber (medical or other). Although only 2% of the nurses in the sample had prescribing rights, all reported some influence, whether it be developing policies or guidelines that involved the use of medications, or making recommendations to medical staff about medication prescribing. Risk management is particularly important in relation to advising on appropriate medication and dosages. Where legal responsibility lies in this situation needs careful consideration and is clearly an area where further exploration could be undertaken to ensure accountability is appropriately acknowledged and visible.These factors suggest the need to examine how the teaching of essential knowledge in the clinical environment is organised within the multidisciplinary team. In midwifery, it has been reported that midwives’ preceptor medical students at the expense of midwifery students.^6^ There may also be conflicts between informal medical education needs and pressure to educate nursing students. Castledine^14^ is in favour of a collaborative style of healthcare practice and sees taking on more responsibility as an inevitable aspect of growing nursing autonomy. He notes that “a truly collaborative interdisciplinary practice model for nurses and physicians is possible as long as both professions operate from a basis of equal power and mutual respect”. Christenson and Hewitt-Taylor^15^ point out that if nurses have the opportunity for additional roles they must make sure that it is in the best interest of patient care and not just to free up medical staff.The study has illuminated a hidden component of nursing and midwifery activities but there are research limitations. Nurses from three countries and midwives from two countries were invited to respond to the NMeS. While nursing numbers for each country are substantial, the findings cannot be extrapolated to nurses and midwives outside the respondent group. The responses to the questions were not mandatory. It may be that only those nurses and midwives who were substantially engaged in teaching doctors, or held a belief that this was part of their role, would have responded. Consequently, response bias may have resulted in overestimation of the extent to which nurses and midwives informally teach medical staff. This study is also based on self-reported responses to a series of questions relating to teaching doctors, so we have no way of validating the responses. Another limitation is that data is drawn from a survey conducted in 2008 and 2009 and there could be changes in medical education during this period which impact on the survey findings. However, anecdotal evidence would support the conclusion that little has changed in the intervening period in terms of the clinical teaching activities of nurses and midwives.This research raises questions and issues that could be usefully explored in further studies. A measure of the actual time spent by nurses and midwives in informal medical education in a range of health care settings is necessary to establish if there are significant workload issues that require recognition and resources. It would also be useful to explore the satisfaction of nurses and midwives with their informal teaching activities.

## Conclusion

Most nurses and midwives in this study self-reported informal contributions to medical education with one-fifth perceiving a moderate to a lot of time spent on medical staff education. Education in the clinical setting is not a unidirectional process. In the context of contemporary nursing and midwifery practice the education of advanced and specialist practitioners (such as nurse practitioners) relies on the support and formal education provided by medical colleagues. In the era of interprofessional education and multidisciplinary health care teams, across discipline teaching is of paramount importance and it is likely that nurses and midwives may assume greater responsibilities in medical education.The implications for medical education arising from this research include the need to examine the models framing medical and nursing entry into clinical practice. A comparison of formalised preceptor models with more informal experiential learning approaches would be useful in ascertaining the impact on clinical priorities and patient care associated with each approach. The recommendations of Lublin and Gething^1^ published in 1992 are also still relevant today. There is a need to examine the type of educational activities that take place between nurses and doctors, and the recognition and resourcing of significant contributions to that education. A clear articulation of teaching activities is the first step to a more formalised development of teaching protocols and supervision responsibilities in areas such as advice about medication prescribing and documentation.

### Acknowledgements

The authors would like to thank Lindy Humphreyes-Reid and Christine Benefer for management of the Nurses and Midwives e-cohort study. The research on which this paper is based was conducted as part of the Nurse and Midwives e-cohort study (www.e-cohort.net), at The University of Queensland. Funding support from the Australian Research Council, the National Health and Medical Research Council, Queensland health and the Department of Health, South Australia. This project is supported by grants from the Australian Research Council (LP0562102), (SR0566924), Australian National Health and Medical Research Council (2005002108) and New Zealand Health Research Council (456163). Industry Partners providing additional funding include: Queensland Health, the South Australian Department of Health, Injury Prevention and Control Australia (Pty Ltd), Nursing Council of New Zealand and the Macquarie Bank Foundation. Industry partners providing in-kind support to the project include: Queensland Nursing Council, Nurses and Midwives Board of New South Wales, Nurses Board of Tasmania, Nurses Board of Western Australia, Nurses Board of the Australian Capital Territory and the Nursing Council of New Zealand, Corporate sponsors include Virgin Blue, Virgin Atlantic and MessageNet.

### Conflict of Interest

The authors declare they have no conflict of interest.
